# Differences in Gene Expression between First and Third Trimester Human Placenta: A Microarray Study

**DOI:** 10.1371/journal.pone.0033294

**Published:** 2012-03-19

**Authors:** Vasilis Sitras, Christopher Fenton, Ruth Paulssen, Åse Vårtun, G. Acharya

**Affiliations:** 1 Department of Obstetrics and Gynecology, Akershus University Hospital, Lørenskog, Norway; 2 Women's Health and Perinatology Group, University of Tromsø and University Hospital of North Norway, Tromsø, Norway; 3 Institute of Clinical Medicine, University of Tromsø, Tromsø, Norway; VU University Medical Center, The Netherlands

## Abstract

**Background:**

The human placenta is a rapidly developing organ that undergoes structural and functional changes throughout the pregnancy. Our objectives were to investigate the differences in global gene expression profile, the expression of imprinted genes and the effect of smoking in first and third trimester normal human placentas.

**Materials and Methods:**

Placental samples were collected from 21 women with uncomplicated pregnancies delivered at term and 16 healthy women undergoing termination of pregnancy at 9–12 weeks gestation. Placental gene expression profile was evaluated by Human Genome Survey Microarray v.2.0 (Applied Biosystems) and real-time polymerase chain reaction.

**Results:**

Almost 25% of the genes spotted on the array (n = 7519) were differentially expressed between first and third trimester placentas. Genes regulating biological processes involved in cell proliferation, cell differentiation and angiogenesis were up-regulated in the first trimester; whereas cell surface receptor mediated signal transduction, G-protein mediated signalling, ion transport, neuronal activities and chemosensory perception were up-regulated in the third trimester. Pathway analysis showed that brain and placenta might share common developmental routes. Principal component analysis based on the expression of 17 imprinted genes showed a clear separation of first and third trimester placentas, indicating that epigenetic modifications occur throughout pregnancy. In smokers, a set of genes encoding oxidoreductases were differentially expressed in both trimesters.

**Conclusions:**

Differences in global gene expression profile between first and third trimester human placenta reflect temporal changes in placental structure and function. Epigenetic rearrangements in the human placenta seem to occur across gestation, indicating the importance of environmental influence in the developing feto-placental unit.

## Introduction

Molecular, histological and functional rearrangements of the placenta are necessary throughout pregnancy in order to ensure appropriate fetal development and maternal health. The gestation-related regulation of placental development is probably driven by genetic, fetal, maternal and environmental factors. However, the molecular mechanisms behind this process are unknown [1]. As gestation advances the needs of the fetus change and the placenta adapts to these changes. Placental weight is related to fetal growth, with progressive increase of the fetal-placental weight ratio from 1∶2.9 at 24 weeks gestation to 1∶6.8 at term [2].

The formation of the placenta is characterised by a rapidly growing, undifferentiated trophoblast that acquires a villous and an extravillous phenotype. The villous cytotrophoblast enters the syncytial pathway, while the extravillous trophoblast invades the maternal decidua. Both lineages differentiate in parallel in order to establish the feto-maternal circulation. The total surface area of the villi in the normal human placenta is linearly associated with placental volume [3]. Morphological studies have demonstrated a continuous evolution of the different chorionic villous types during gestation [4]. The decrease in trophoblast proliferation along with a relative increase in endothelial proliferation causes a switch from branching to non-branching angiogenesis in the third trimester. This results in the formation of long and slender villous trees containing one or two poorly branched capillary loops, which in turn, cause a decrease in feto-placental vascular impedance [5]. These morphological changes in villous tree development are reflected in the hemodynamic changes observed in the feto-placental circulation. Indeed, ultrasound assessment of the feto-placental circulation shows a gradual increase in the fraction of the fetal cardiac output distributed to the placenta in the second trimester [6] with slight decrease towards term [7].

Intrauterine environment influences placental development. There is a switch from histiotrophic nutrition in the first trimester to hemotrophic nutrition later in pregnancy [8]. Moreover, *in utero* environment seems to play an important role not only for fetal development, but also for health in adult life, through epigenetic programming. The placenta is considered to be a major site of epigenetic regulation from the pre-implantation period to delivery [9].

The human placenta expresses more than 12000 genes [10], including most of the currently known imprinted genes. We hypothesized that molecular rearrangements and phenotypic adaptations that are necessary for normal placental development are reflected in its gene expression levels. The aims of this study were to investigate differences in global gene expression profile, the expression of imprinted genes in particular and the effect of smoking in the first and third trimester placenta during normal human pregnancy.

## Results

The phenotype of the study population is shown in Table 1. There were no differences regarding maternal age, gravidity and parity among groups, but the percentage of women smoking tobacco was higher in the first trimester group compared to the third trimester.

**Table 1:** Phenotype of the study population.

	I trimester (n = 16)	III trimester (n = 21)	p-value
Maternal age (years)	28.19±6.8	30.24±4.8	0.3
Gravidity (n ± standard error)	3.3±0.5	2.9±0.6	0.5
Parity n (n ± standard error)	1±0.3	1.3±0.5	0.7
Smoking n (%)	9 (56)	3 (14)	0.01
Gestational age at sampling (days)	71.2±8	275±8	0.00

Data are presented as mean ± SD, n (%) or median (range). Differences between the groups were assessed using Student's t-test for parametric and Chi-square test for categorical variables.

**Figure 1:**
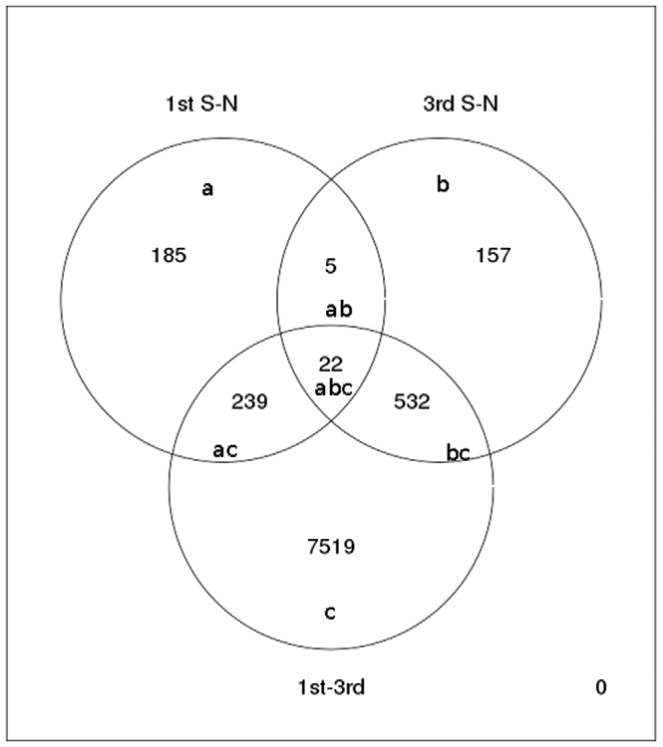
Venn diagram showing the number of differentially expressed genes between the groups: smoking versus non-smoking placentas at the first (a) and third (b) trimester of pregnancy; and first versus third trimester placentas irrespective of smoking status(c). The areas of interception of the circles (ab+abc) indicate 27 genes that were affected by smoking throughout pregnancy.

**Figure 2:**
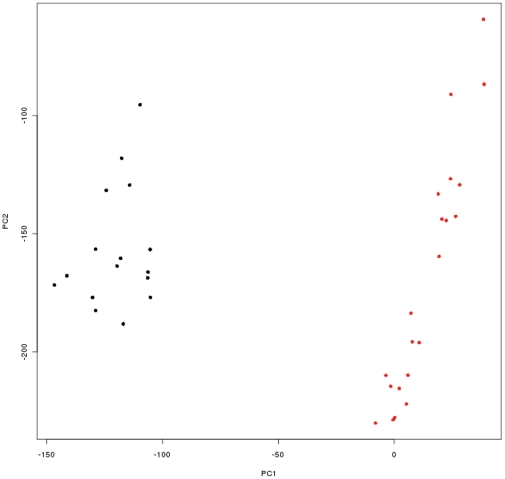
Two-dimensional principal component analysis of the differentially expressed placental genes with best t-scores in the main analysis. Each spot represents a placental sample: 16 first trimester placentas (black spots) and 21 third trimester placentas (red spots).

**Figure 3:**
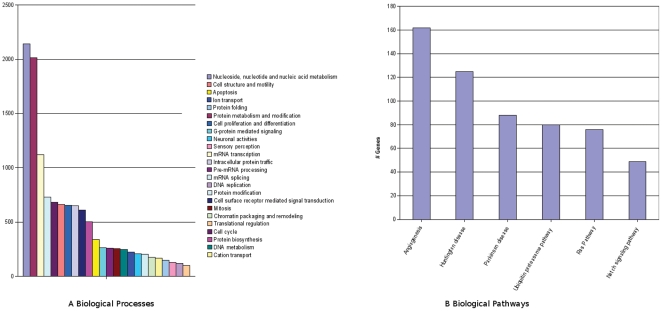
Box plots of the biological processes (3a) and molecular pathways (3b) that were differentially expressed between first and third trimester placentas. The x axis represents the number of genes involved in each process/pathway that were differentially expressed between the groups.

We found 7519 genes to be differentially expressed between first and third trimester placentas (Figure 1 and TableS1), representing almost 25% of the genes spotted on the array. Principal component analysis (PCA) showed a clear separation between first and third trimester placentas (Figure 2). Panther analysis with Bonferroni correction for multiple testing (p≤0.01) showed several biological processes (Figure 3a) and molecular pathways (Figure 3b) to be differentially expressed between the groups. Among differentially expressed genes, those involved in biological processes such as nucleic acid metabolism, protein metabolism and modification, mRNA transcription, cell cycle, cell structure and motility were highly (p≤0.001) up-regulated in the first trimester placentas, whereas cell surface receptor mediated signal transduction, G-protein mediated signalling, ion transport, neuronal activities and chemosensory perception were up-regulated in the third trimester. Pathway analysis indicated that genes involved in angiogenesis, Huntington disease, Parkinson disease, Ubiquitin proteasome, Ras and Notch signalling pathways were differentially expressed between first and third trimesters. We used Human Neurogenesis and Neural Stem Cell PCR Array to test the hypothesis that brain and placenta might share common developmental pathways. We found that twenty six out of the 84 genes (31%) represented on the PCR array that are known to be involved in human neurogenesis were significantly (i.e ≥2 fold) differentially expressed between first and third trimester placentas (Figure S1). Of these 26 genes, 12 showed good correlation with PCR, 9 genes had low expression levels in microarrays, 1 gene was not differentially expressed between the groups in microarrays and 4 genes had opposite expression in microarrays compared to PCR (Table S2).

We further investigated gene expression profiles of 50 known imprinted human genes between first and third trimester placentas. Many of these genes were not present on the array or had very low intensity signals. However, PCA based on the expression of 17 imprinted genes could clearly differentiate first and third trimester placentas (Figure S2). We validated this result by RT-PCR for 6 selected imprinted genes and found that insulin-like growth factor 2 (IGF2) and Pleckstrin homology-like domain family A member 2 (PHLDA2) were differentially expressed between the groups (Table S3).

A subgroup analysis showed that 27 genes were differentially expressed in the placentas of the women who smoked during pregnancy compared to non-smokers, both in the first and third trimesters of pregnancy (Figure 1). Four among the 27 genes that were affected by smoking encode for oxidoreductases. More specifically cyclooxygenase 2 and 5B (COX2, COX5B) and cytochrome P450 (CYP) 2D1 and −2D7 isoforms (CYP2D1, CYP2D7) were differentially expressed both in the first and third trimester placentas from women smoking during pregnancy. We validated this result by PCR on individual placental samples and found that COX2 was up-regulated and COX5B was down-regulated by smoking in both trimesters (Table S4).

## Discussion

There is abundant literature regarding differential gene expression profile between healthy and compromised placentas, specially in the context of preeclampsia [11]. However, differences in gene expression profile during normal development of the human placenta are still not fully understood. Previously only one microarray study has addressed this issue [12]. Our aim was to find molecular pathways involved in gestation-related physiological changes in placental structure and function. We found that more than half of the genes that are expressed in the human placenta -i.e. 7519 of 12000 genes expressed in placenta - change their expression profile from the first to the third trimester of pregnancy, which confirms that the placenta undergoes a profound molecular rearrangement in order to adapt to the changing demands of the fetus.

Genes related to biological processes such as nucleic acid metabolism, protein metabolism and modification, mRNA transcription, cell cycle, cell structure and motility were highly expressed in the first trimester placentas indicating that the placenta undergoes intense cell proliferation and differentiation during this period. This observation is in accordance with the findings of Mikheev et al [12] and is also supported by another genomic study on human placenta [13]. Longitudinal studies on gene expression in human placenta are lacking mainly due to ethical constraints. However, a study in mice suggested that a transition occurs at mid-gestation and different genes are expressed by the same cellular populations without any major morphological changes in the placenta [14]. Moreover, a human microarray study of basal plate biopsy specimens of the maternal-fetal interface showed dramatic changes between midgestation and term [15]. This is in line with the pattern of fetal and placental weight gain seen in human pregnancy. In early gestation the weight of the placenta is higher and increases more rapidly than the weight of the fetus, but the placental growth slows down later in gestation while that of the fetus continues with an apparent cross-over around mid-gestation.

Pathway analysis of the differentially expressed genes between first and third trimester placentas showed angiogenesis to be highly active in first trimester. Other molecular pathways that were up-regulated in the first trimester placentas are involved in neurodegenerative disorders, namely Huntington disease, Parkinson disease and Notch signalling pathway. Our results from the Neurogenesis and Neural Stem Cell PCR Array showed an overall good correlation between microarray and PCR (Table S2). This is in accordance with Morey J. et al. [16] who found that genes exhibiting at least 1.4 fold change and p-value of ≤0.0001 in microarray analyses consistently yield significant correlations of at least 0.80 for array and PCR data. Furthemore, our results indicate that there might be common developmental pathways between brain and placenta. Indeed stem cells derived from human placenta can be differentiated into neural progenitors *in vitro* [17]. Moreover, the placenta produces a wide range of neurohormones [18] that might affect fetal brain development [19]. In a previous study we found similar neurologic molecular pathways to be up-regulated in placentas affected by preeclampsia [20]. One can thus hypothesize that misregulation of these molecular pathways in early pregnancy could result in early pregnancy loss, preeclampsia or IUGR.

Imprinting is an important genetic phenomenon occurring in mammals [21]. Many genes undergoing epigenetic modifications are expressed and specifically imprinted in the human placenta [22]. Placental imprinting seems to be a dynamic process occurring throughout pregnancy [23]. Using whole genome methylation data in first and third trimester placental tissue, a methylation-induced down regulation has been demonstrated for a set of tumour-associated genes, as part of normal placentation [24]. We found that expression of imprinted genes occurs in a temporal manner during normal human placental development. We demonstrated that IGF2 and PHLDLA2 were differentially expressed between first and third trimester placentas. Unfortunately, we are not able to include allele-specific analysis of heterozygous samples because we did not collect maternal and paternal blood samples. However, according to Monk et al [25] who assessed the imprinting status of placental-specific imprinted genes using first and third trimester placental tissue with corresponding maternal and –for the third trimester only- paternal blood samples, the expression of these genes is largely biallelic throughout pregnancy. It has been shown that epigenetic modifications of these genes have direct effects on placental size and morphology as well as placental transport capacity [26]. Environmental conditions can also alter the epigenetic status of the placenta which in turn affects fetal growth and development [26]. Moreover, several studies have shown that epigenetic disturbances can be associated with placenta-related disorders such as IUGR and preeclampsia.

It is known that smoking has significant morphological and functional effects on the placenta [27]. Several studies have investigated the impact of maternal smoking on placental gene expression at term [28], [29]. These studies have indicated that several genes encoding for xenobiotic-metabolising enzymes of the CYP complex are up-regulated by smoking in a temporal manner. Interestingly, fetal exposure to tobacco increases placental CYP1A1 expression, through hypomethylation of its gene transcription factor binding element [30]. Moreover, Mikheev et al have indicated that the expression of several CYPs is regulated in the normal palecnta of non smoking mothers depending on gestational age, probably because these enzymes are also involved in steroid metabolism [12]. Therefore we performed subgroup analysis in placentas obtained from women who smoked in the first (n = 9/16, 56%) and third (n = 3/21, 14%) trimester compared to non smokers. Although there was an unbalanced proportion of smokers between the groups, data analysis showed that 793 (ac+abc+bc in figure 1) of 7519 (9.5%) differentially expressed genes were affected by smoking. This indicates that the effect of smoking on global placental gene expression profile is limited compared to the effect of placental maturation. Furthermore, we found in microarrays that only 27 (ab+abc in figure 1) placental genes were affected by smoking both in the first and third trimester; four of which are oxidoreductases. PCR validation of these genes showed specific regulation of COX2 and COX5B by smoking regardless of gestational age. Moreover, there is epidemiologic evidence that maternal cigarette smoking during pregnancy reduces the risk of developing preeclampsia in both primiparous and multiparous women [31], [32]. Interestingly, we previously found several oxidoreductases to be differentially expressed between preeclamptic and normal placentas (suppl table 1 in reference 20). It seems therefore reasonable to assume that the regulation of specific xenobiotic enzymes from early pregnancy until term in smoking mothers might protect them against preeclampsia.

In conclusion, we found profound differences in global gene expression profile between first and third trimester human placenta reflecting temporal changes in placental structure and function. Epigenetic rearrangements in the human placenta seem to occur across gestation, indicating the importance of environmental influence in the developing feto-placental unit. Smoking affects specific placental oxidoreductases throughout pregnancy.

## Methods

### Study population

We recruited 24 healthy women with uncomplicated pregnancies that were delivered at term and 16 women undergoing surgical termination of pregnancy for social reasons, between 9–12 weeks of gestation. All women were healthy, Caucasian and had low risk pregnancies. Gestational age was assigned by abdominal ultrasound at 18–20 weeks of gestation and by transvaginal ultrasound before termination of pregnancy. All pregnant women recruited to the study had a physical examination and ultrasonography ≤48 hours before delivery or termination of pregnancy. The study was approved by the Regional Ethics Committee for Medical Research - North Norway (REK-Nord 94/2004) and informed written consent was obtained from all participants.

### Placental sample collection and conservation

Third trimester placental samples were obtained immediately after delivery. Chorionic tissue was dissected from a standardized location (approximately 2 cm beside the umbilical cord insertion, from the middle layer of placenta midway between maternal and fetal surfaces). Placental samples were collected from macroscopically normal areas excluding sites of infarction, haemorrhage and fibrin deposition. Chorionic tissue was obtained immediately after termination of pregnancy from first trimester placentas and was separated from decidua using light microscopy. The collected specimen was transferred to a Petri dish and washed thoroughly with physiological saline to remove any contamination with maternal blood and amniotic fluid. Each tissue sample was transferred to tubes containing 1.5 ml RNA*later* solution (RNA stabilization reagent, Qiagen GmbH, Germany), and stored at −70°C until RNA isolation, microarray experiment and RT-PCR was performed.

### RNA isolation and quality/quantity control

Disruption and homogenization of tissue specimens were performed in lysis buffer using the MagNa Lyser Instrument (Roche Applied Science, Germany), according to the manufacturer's instructions. Isolation of total RNA was performed using the MagNa Pure Compact RNA isolation kit and the MagNa Pure Compact Instrument (Roche Applied Science, Germany). RNA was quantified by measuring absorbance at 260 nm, and RNA purity was determined by the ratios OD260 nm/280 nm and OD230 nm/280 nm using the NanoDrop instrument (NanoDrop^®^ ND-1000, Wilmington, USA). The RNA integrity was determined by electrophoresis using the Agilent 2100 Bioanalyser (Matriks, Norway). Only samples with RNA Integrity Number (RIN) >7.2 were used for microarray.

### Microarray experimental design

mRNA was extracted for hybridization from 24 third trimester and 16 first trimester placental samples. Three third trimester placentas were excluded due to RIN<7.2. A total of 37 hybridisations were performed applying a direct comparison design.

### Microarray procedures

Total RNA samples were processed into digoxigenin (DIG)-labelled cRNA using the Applied Biosystems Chemiluminescent NanoAmp™ RT-IVT Labeling Kit. The labelled DIG-cRNA (10 µg per microarray) was then injected into each microarray hybridization chamber. Following hybridization at 55°C for 16 hours, the unbound material was washed from the microarrays. Features that retained bound DIG-labelled cRNA were visualized using the Applied Biosystems Chemiluniescence Detection Kit. Anti-DIG alkaline phosphatase was used to hydrolyse a chemiluminescence substrate to generate light at 458 nm which was than detected by the Applied Biosystems 1700 Chemiluninescent Microarray Analyzer. The Human Genome Survey Microarray v.2.0 (Applied Biosystems) with 32878 probes for the interrogation of 29098 genes was used for microarray analysis.

### Data processing and statistical analysis

Microarrays were scanned and preprocessed using the R bionconductor package (http://www.r-project.org) (scripts attached in Table S5). Probes with unusual signal patterns were flagged by the scanner and signal strength set to NA (not a number). Arrays showing low correlation between hybridization controls were removed. Missing intensity values were imputed using the nearest neighbour method [33]. The resultant intensity matrix was normalised by quantile normalization [34]. Probes with a log2 average signal less than 8 and a variance less than 0.1 were removed as background. PCA was performed on the remaining probes. Principal component 1 accounted for 27% of cumulative variance, and principal component 2 for 10% of the cumulative variance.

### Gene annotations

We used Protein ANalysis THrough Evolutionary Relationships (PANTHER) [35] which is a freely available, comprehensive software system for relating gene sequence to specific molecular functions, biological processes and pathways (http://www.pantherdb.org). The expression data analysis tool, with Bonferroni correction for multiple testing, was used in order to find signalling pathways that might be involved in normal placental development.

### Database submission of microarray data

The microarray data were prepared according to minimum information about a microarray experiment (MIAME) recommendations [36] and deposited in the Gene Expression Omnibus (GEO) database: http://www.ncbi.nlm.nih.gov/geo/. The GEO accession number for the platform is GSE28551, samples GSM 707051-GSM 707087.

### Validation of microarray results by PCR-array and RT-PCR

We carried out quantitative RT-PCR with 200 µg total RNA isolated from a pool of first and a separate pool of third trimester chorionic tissue, using RT^2^ ProfilerTM Human Neurogenesis and Neural Stem Cell PCR Array (PAHS-404, SABiosciences Corporation, USA) according to the manufacturer's instructions. The plate contains primers for 84 genes related to the regulation of key neurogenesis processes such as the cell cycle and cell proliferation, differentiation, motility, and migration. This array contains the growth factors and cytokines related to neural stem cells as well as genes involved in synaptic functions, apoptosis and cell adhesion. For detailed layout of the array see http://www.sabiosciences.com/rt_pcr_product/HTML/PAHS-404A.html.

Actin beta (ACTB) was used as housekeeping gene. Analysis of fold changes was done by the ΔΔCt method using the integrated web-based software package for the PCR Array System.

Further, the expression of selected six imprinted genes was validated by RT-PCR. The selection was based on: a) their colour intensity on the heat map and b) existing information in the literature on these genes regarding their role in placental development and disease. We chose to perform RT-PCR for each of these genes on every individual placenta in order to find differences in expression between the two trimesters, as well as to explore the range of normal variations in expression within samples in the same trimester. This method is more accurate, although it necessitates more mRNA.

Total RNA was reverse transcribed using iScript cDNA Synthesis Kit (Bio-Rad Laboratories, Cat# 170-8891) as described by the manufacturer's protocol. RT- PCR amplification was performed with an ABI HT7900 Instrument (Applied Biosystems) using the FastStart TaqMan Probe Master [Rox] (Roche, Cat. No. 04673468001) with addition of Rox Reference Dye (Roche, Cat. No. 04673549001). Thermal cycling conditions were as follows: denaturation for 10 minutes at 95°C, then 40 cycles PCR with denaturation for 15 seconds at 95°C; annealing and extension for 1 minute at 60°C. Sample volume used was 20 µL. Primers and probes were constructed by using Universal ProbeLibrary program (Roche), given in Table S6. The primers were synthesized and purified by Thermo Fisher Scientific (Germany) and the primers for the housekeeping genes by Eurogentec S.S. (Belgium). The probes were obtained from Universal ProbeLibrary Probes 1–165 (Roche). Cyclophylin A and hypoxanthine phosphoribosyl-transferase 1 (HPRT) were used as reference genes. Samples for each experiment were run in duplicate and averaged for final quantification. The fold inductions were calculated as described previously [37].

## Supporting Information

Figure S1 **Scatter plot of the log transformed expression ratio of all 84 genes spotted on the RT2 ProfilerTM Human Neurogenesis and Neural Stem Cell PCR Array.** Each spot represents a gene. The colour represents the level of gene expression: red indicates up-regulation in the third trimester (group 1); green indicates up-regulation in the first trimester (control group). The black spots represent genes that had a relative expression of ≤2 fold between the groups.
(JPG)

Figure S2 **Two-dimensional principal component analysis of the differentially expressed placental imprinted genes.** Each spot represents a placental sample: 16 first trimester placentas (black spots) and 21 third trimester placentas (red spots).
(JPG)

Table S1 **List of all the genes, including fold-change and p-value, which were present on the arrays.**
(XLS)

Table S2 **Expression of the 26 genes that had a fold change of ≥2 in Neurogenesis PCR array and their relative expression (fold-change and p-value) in microarrays.**
(XLS)

Table S3 **Expression of selected imprinted genes (IGF2, PHLDA2, PLAGL1, SNPRN and SLC22A18) in individual placental samples, in the first and third trimester of pregnancy.**
(XLS)

Table S4 **Expression of COX2, COX5B, CYP2D1 and CYP2D7 in placental samples obtained from women who smoked tobacco compared to women who did not smoke tobacco throughout pregnancy.**
(XLS)

Table S5 **R bionconductor scripts used for processing microarray data.**
(TXT)

Table S6 **Primers and probes used for RT-PCR validation.**
(DOC)
