# Supplementary figures and images for: Differences in Gene Expression between First and Third Trimester Human Placenta: A Microarray Study
Source: PLoS One. 2012 Mar 19;7(3):e33294. doi: 10.1371/journal.pone.0033294 (PMC3307733; doi:10.1371/journal.pone.0033294)

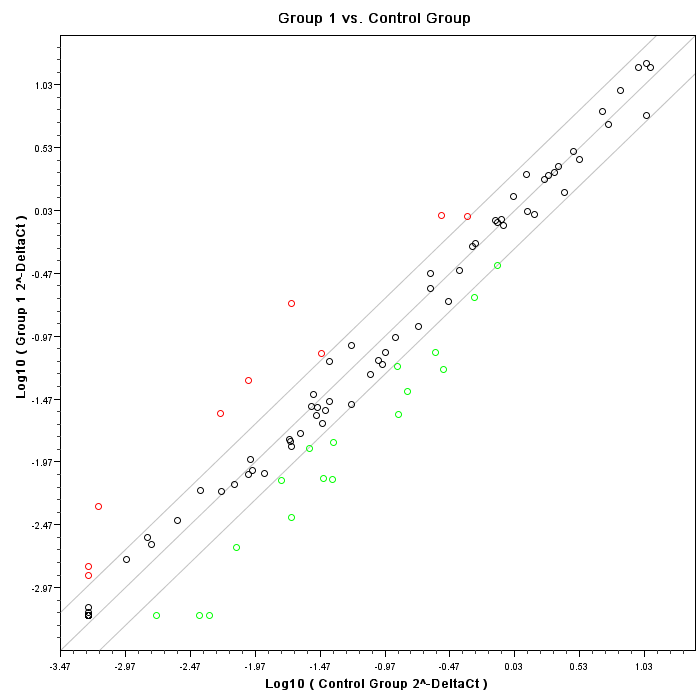

Supplement: Figure S1 — Scatter plot of the log transformed expression ratio of all 84 genes spotted on the RT2 ProfilerTM Human Neurogenesis and Neural Stem Cell PCR Array. Each spot represents a gene. The colour represents the level of gene expression: red indicates up-regulation in the third trimester (group 1); green indicates up-regulation in the first trimester (control group). The black spots represent genes that had a relative expression of ≤2 fold between the groups. (JPG) [file pone.0033294.s001.jpg]

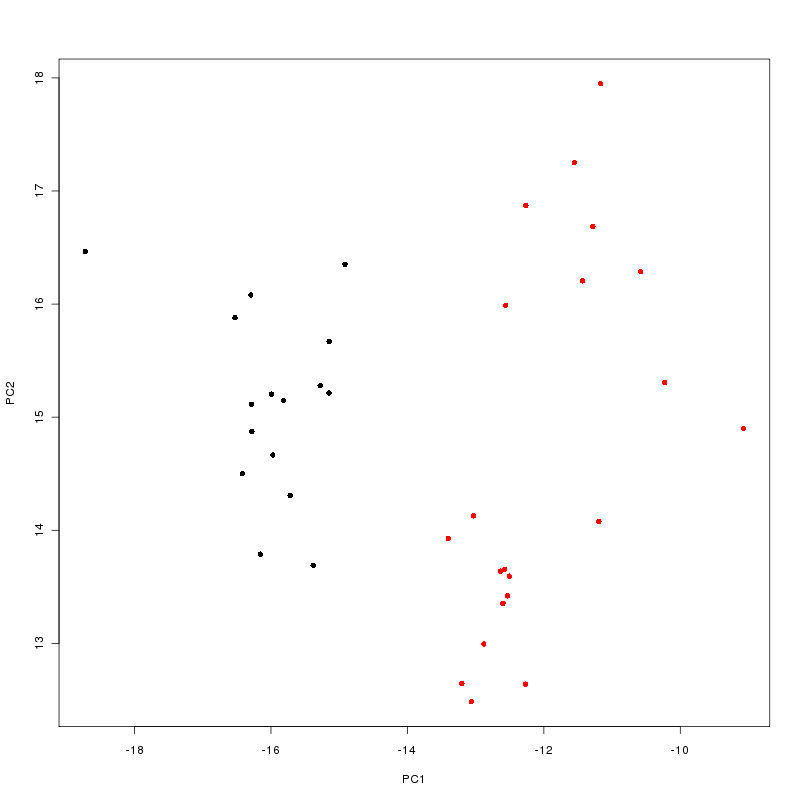

Supplement: Figure S2 — Two-dimensional principal component analysis of the differentially expressed placental imprinted genes. Each spot represents a placental sample: 16 first trimester placentas (black spots) and 21 third trimester placentas (red spots). (JPG) [file pone.0033294.s002.jpg]
