# Supplementary material for: Differences in Gene Expression between First and Third Trimester Human Placenta: A Microarray Study
Source: PLoS One. 2012 Mar 19;7(3):e33294. doi: 10.1371/journal.pone.0033294 (PMC3307733; doi:10.1371/journal.pone.0033294)
Supplement: Table S6 — Primers and probes used for RT-PCR validation. (DOC) [file pone.0033294.s008.doc]

**Table S6:** Primers and probes used for RT-PCR validation

| **Primer** | **ProbeFinder** | **Sequence** | **Universal ProbeLibrary** |
| --- | --- | --- | --- |
| **IGF2, left** | NM_000612.4 | acaccctccagttcgtctgt | **# 40** |
| **IGF2, right** |  | gaaacagcactcctcaacga |  |
| **PLAGL1, left** | NM_001080951.1 | cagcagatgttccctgtcac | **# 68** |
| **PLAGL1 right** |  | gggagaaagtctgaggcaca |  |
| **SNRPN left** | NM_003097.3 | gaggagttgggggaccat | **# 62** |
| **SNRPN right** |  | cagctgctacagtgcctcttc |  |
| **PHLDA2 left** | NM_003311.3 | acagcctcttccagctatgg | **# 58** |
| **PHLDA2 right** |  | ctccacgcagtccacctt |  |
| **SLC22A18 left** | NM_183233.2 | tgcttcacctggaggaagac | **# 27** |
| **SLC22A18 right** |  | caggggtgctgagtcactg |  |
| **ZNF331 left** | NM_018555.5 | ggcctgtctgaactctgctc | **# 67** |
| **ZNF331 right** |  | tcatatgctgactccaaatcca |  |
| **Cyclophylin A left** | CR456707.1 | tgctggaccaacacaaat | **# 48** |
| **Cyclophylin A right** |  | cacatgcttgccatccaa |  |
| **HPRT left** | NM_000194 | ggactgacactggcaaaacaatgca |  |
| **HPRT right** |  | agcttgcgaccttgaccatct |  |
| **HPRT probe** |  | ttgctttccttggtcaggcagtataatcca |  |

|  |  |  |  |
| --- | --- | --- | --- |
| **ACOX2 Fw** | NM_003500.3 | **cagaaggcctgtgtcatcg** | **#70** |
| **ACOX2 Rev** |  | **agtccaggacctttgcctct** |  |
| **COX5B Fw** | NM_001862.2 | **tccatggcatctggaggt** | **#71** |
| **COX5B Rev** |  | **tgtatgggtccagtcccttct** |  |
| **CYP2D6 Fw** | NM_001025161.1 | **aggaggagtcgggctttct** | **#56** |
| **CYP2D6 Rev** |  | **cgctgggatatgcaggag** |  |
| **CYP2D7P1 Fw** | NM_002570.2 | **aaggaggagtcgggcttc** | **#56** |
| **CYP2D7P1 Rev** |  | **tttggaagcgtaggaccttg** |  |
